# Bridging pDCs and cDCs: The Identity of Transitional Dendritic Cells

**DOI:** 10.1111/imr.70070

**Published:** 2025-11-23

**Authors:** Juliana Idoyaga, Hai Ni, Raul A. Maqueda‐Alfaro

**Affiliations:** ^1^ Department of Pharmacology University of California San Diego School of Medicine La Jolla California USA; ^2^ Department of Molecular Biology University of California San Diego School of Biological Sciences La Jolla California USA

**Keywords:** cDC2A, cDC2B, conventional dendritic cells (cDC), IL‐1β, plasmacytoid dendritic cells (pDCs), transitional dendritic cells (tDCs)

## Abstract

Transitional dendritic cells (tDCs) have emerged as a compelling addition to the dendritic cell (DC) network—a hybrid subset that bridges plasmacytoid (pDC) and conventional (cDC) lineages, particularly conventional type 2 DCs (cDC2s). First identified through high‐dimensional single‐cell profiling, tDCs combine features of both pDCs and cDC2s yet follow a distinct developmental trajectory with unique effector functions. Although ontogenetically related to pDCs, tDCs do not produce type I interferon but instead mount a robust IL‐1β response upon pathogen sensing, positioning them as rapid initiators of innate inflammation. tDCs also mirror cDC2s in their ability to capture antigen and prime naïve CD4^+^ T cells. Importantly, tDCs exist in progressive states—tDC^lo^, tDC^hi^, CD11b^−^ tDC2s and tDC‐derived DC2s (tDC2s)—reflecting a unidirectional differentiation continuum. Recognizing this dynamic spectrum is essential for properly interpreting tDC function and avoiding fragmented nomenclature. In this review, we synthesize current insights into tDC biology across species—tracing their origin, phenotypic and transcriptional trajectory, tissue localization, and immune function. Although tDCs challenge the rigid pDC/cDC dichotomy, they exemplify a broader principle: DC identity is not fixed but temporally programmed, even during homeostasis. Embracing this plasticity may unlock new opportunities for therapeutic intervention in infection, cancer, and autoimmunity.

## 1 Introduction

Understanding the functional diversity of dendritic cells (DCs)—manifested through distinct subsets and states—is fundamental to decoding the complexity of immune responses elicited by a broad range of triggers. DC subsets were originally defined by the expression of a limited set of surface markers, such as CD8, CD205/DEC‐205 and CD11b [1, 2], which are often nonspecific and variably expressed across tissues and species. Consequently, a nomenclature based mainly on developmental origin was proposed [3]. This framework grouped DCs into conventional DCs (cDCs)—which can be further divided into type 1 (cDC1) and type 2 (cDC2) subsets with enhanced capacity to prime T cells—and plasmacytoid DCs (pDCs), specialized in type I interferon (IFN‐I) secretion in response to viral infection. Because this classification was applicable across tissue types and species [4], it was rapidly adopted by the field and provided a unifying nomenclature for DC biology.

The advent of high‐dimensional single‐cell technologies—such as single‐cell transcriptomic, epigenomic, and proteomic platforms—has dramatically expanded our ability to dissect cellular heterogeneity [5]. When combined with new lineage‐tracing mouse models and adoptive transfer experiments, these tools revealed a previously underappreciated diversity within the DC compartment, uncovering distinctions in lineage potential, transcriptional and phenotypic features, and functional specialization across subsets. Among the most compelling findings to emerge from these approaches was the identification of transitional dendritic cells (tDCs)—also referred to as human AXL^+^ DCs and AXL^+^ SIGLEC6^+^ DCs (ASDCs) [6, 7, 8], or mouse pDC‐like cells [9]. These cells exhibit a hybrid transcriptome, epigenome, and phenotype, expressing gene programs and markers associated with both pDCs and cDCs, particularly cDC2s.

Importantly, tDCs meet several criteria for a bona fide DC subset: they follow a distinct developmental trajectory and exhibit transcriptional and functional features that distinguish them from both pDCs and cDCs across species. Although developmentally related to pDCs, tDCs differ functionally: they sense viruses and their mimics, but—unlike pDCs—do not secrete significant levels of type I interferon (IFN‐I). Instead, they produce IL‐1β. Crucially, tDCs exist in a continuum of transcriptional, phenotypic and functional states. This continuum reflects their plasticity and gradual differentiation, which are often operationally simplified into two subpopulations: tDC^lo^/pDC‐like and tDC^hi^/cDC‐like cells. In addition, tDCs give rise to a subpopulation of cDC2‐like cells expressing CD11b^+^ Esam^+^ Cx3cr1^−^ (here referred to as tDC‐derived DC2s or tDC2s), following a unidirectional differentiation trajectory that proceeds through a CD11b^−^ tDC2 intermediate and gradually acquires enhanced antigen‐processing and presentation capabilities. This developmental output has led some to classify tDCs as cDC2 precursors or pre‐cDC2s [10, 11, 12]. However, emerging data support the concept that tDCs are distinct not only from pDCs and cDC2s, but also from canonical pre‐cDC2s. Rather, tDCs represent a unique immune cell type defined by intrinsic effector functions and a context‐dependent potential to generate cDC2‐like progeny.

In this review, we aim to summarize current knowledge of tDCs, including their discovery, the range of names used to describe them, developmental origin, functional features, and cross‐species alignment. We highlight how these cells serve as a striking example of functional versatility within the DC compartment, representing a subset with distinct molecular and functional identity that nonetheless exists in progressive states and ultimately differentiates into tDC2s [13]. tDCs challenge the rigidity of the cDC1/cDC2/pDC framework and underscore the need to move beyond static subset definitions toward a more flexible conceptual model that accounts for developmental origin as well as dynamic transitions in cell state—that is, the capacity of a single cell to acquire different, transcriptionally regulated programs over time. Because steady‐state tDCs follow a distinct model of versatility, they represent a new frontier in DC biology: they illustrate how developmental history and state transitions intersect to produce sequential, layered immune functions with significant clinical implications. Understanding how these functions are regulated may open new avenues for immunomodulatory strategies in infection, autoimmunity, and cancer.

## 2 The Discovery

It is perhaps not surprising that tDCs were first identified in humans. Human studies often rely on limited sample material, which has driven the early adoption of high‐dimensional single‐cell approaches capable of extracting maximal information from minimal input. These technologies have enabled a more granular dissection of DC heterogeneity than was previously possible using conventional biaxial flow cytometry‐based methods.

The adoption of these unbiased, high‐dimensional profiling tools in blood and human tissues revealed a distinct subgroup of DCs, characterized by the expression of AXL and SIGLEC6 at both the transcriptional (scRNA‐seq) and protein (CyTOF) levels, and consequently termed AXL^+^ SIGLEC6^+^ DCs (ASDCs) or AXL^+^ DCs [6, 7]. A similar population was also described by See et al. [12] and designated as cDC precursors (pre‐cDCs) based on their ability to differentiate into cDCs upon ex vivo culture (see Section 8).

**FIGURE 1:**
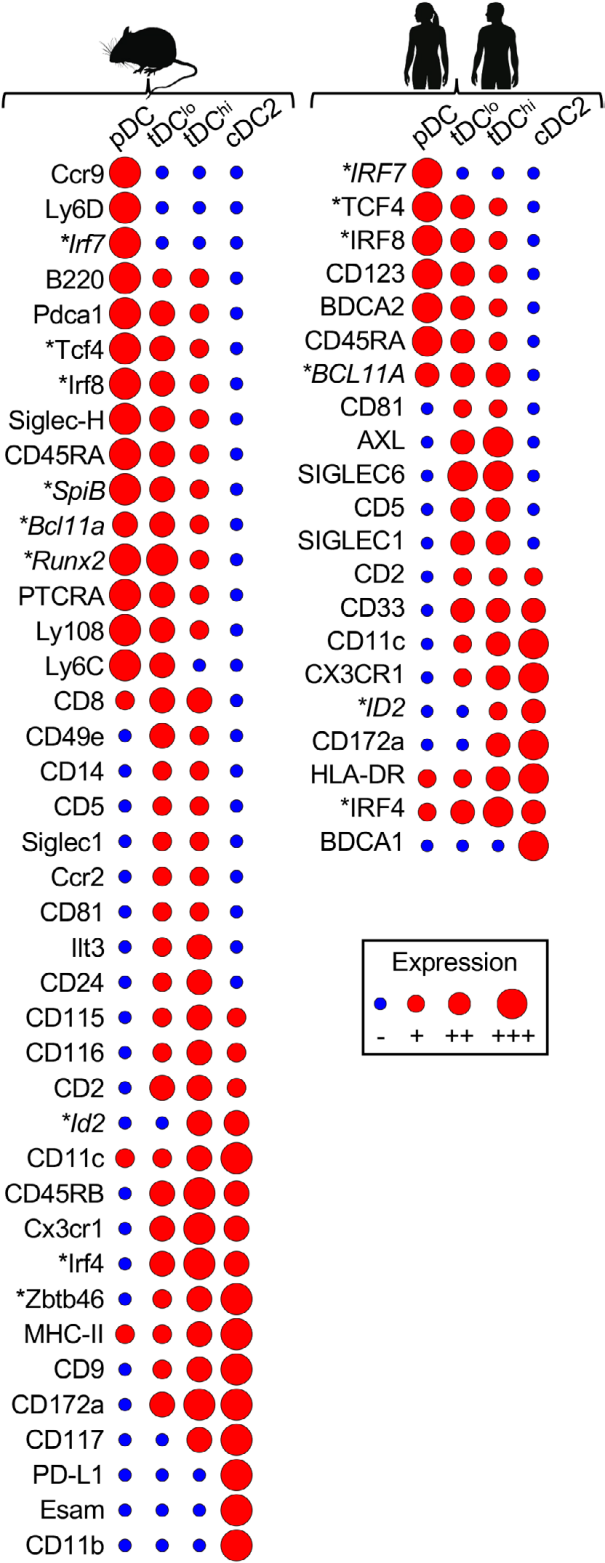
Comparative protein expression profiles of tDCs versus pDCs and cDC2s. Schematic representation of protein expression patterns in tDCs compared with pDCs and cDC2s in both humans and mice. Expression levels indicate qualitative trends rather than experimental measurements. Protein expression data were compiled from studies employing flow cytometry, spectral flow cytometry, or mass cytometry (CyTOF). RNA expression (in italic) was compiled from studies employing sc‐RNAseq, bulk‐RNAseq, or Prime Flow. Data were compiled from studies analyzing human [6, 7, 8, 14, 15] and mouse [8, 10, 14, 16, 17] DCs. Gene and protein nomenclature follows standard conventions: human genes are uppercase italic (e.g., *TCF4*), mouse genes are capitalized italic (e.g., *Tcf4*); proteins are written in the same case, not‐italicized. Asterisks denote TFs.

Unsupervised dimensionality reduction of transcriptomic and protein expression consistently placed ASDCs at the intersection of pDCs and cDC2 clusters, bridging both populations and exhibiting a spectrum from pDC‐like to cDC2‐like features (Figure 1). Single‐cell RNA‐seq analyses showed that ASDCs expressed a continuum of genes and regulatory programs associated with pDCs (e.g., *IL3RA, TCF4, IRF8, IGJ, NRP1, MZB*) and cDC2s (e.g., *ITGAX, LYZ, LY86*) [6]. CyTOF analyses of protein expression revealed that ASDCs exhibited a gradient of surface marker expression, with some cells expressing high levels of pDC markers (e.g., CD123 and BDCA‐2) and others expressing high levels of cDC markers (e.g., CD11c and CD33) [7, 8].

Initial functional screening immediately revealed key differences between ASDCs and pDCs or cDCs. Unlike pDCs, ASDCs produced negligible levels of type I interferons in response to TLR7/8 and TLR9 (CpG‐A and ‐C) agonists [6, 8, 12]. Instead, they exhibited enhanced capacity to activate allogeneic T cells—a functional profile more aligned with cDCs [6, 7, 12].

Altogether, these studies identified a distinct population of human DCs with transcriptomic, phenotypic, and functional characteristics that challenge the classical boundaries of pDCs and cDC2s.

## 3 The Interspecies Alignment

Following their discovery, it remained unclear whether ASDCs were unique to humans or conserved across species. Thus, a parallel comparative multi‐dimensional analysis of human and mouse DCs was necessary to reconcile the findings and identify the murine homolog of these DCs. CyTOF and transcriptomic analyses of the mouse spleen enabled the identification of a DC population that bridges pDCs and cDC2s, similar to what was observed in humans [8]. In mice, this bridging population expressed high levels of Cx3cr1 and variable levels of CD11c, CD33, CD2, CD5, CD81, and Siglec‐1 (CD169) (Figure 1)—all of which are also expressed by human ASDCs [6, 8]. However, the two canonical markers of human ASDCs, AXL, and SIGLEC6, appear to be species‐specific: AXL expression is only observed in humans at steady state, and SIGLEC6 is not conserved in mice. To reflect their shared hybrid identity across species, without relying on species‐specific markers, we proposed the unified term *transitional dendritic cells (tDCs)* [8]. This designation emphasizes their intermediate phenotype and transcriptional profile, conserved in both mouse and human, while avoiding reliance on nonshared surface markers.

Similar to humans, mouse tDCs clustered separately from cDC1s in unsupervised dimensionality reduction maps and lacked expression of cDC1‐specific markers, such as Xcr1 and Clec9a [8]. Like human tDCs, mouse tDCs exhibited substantial internal heterogeneity, forming a phenotypic and transcriptional continuum of features between pDCs and cDC2s. Accordingly, in both species, tDCs could be divided into two populations based on CD11c expression: CD11c^low^, referred to as tDC^lo^, which are more similar to pDCs; and CD11c^high^, referred to as tDC^hi^, which are more similar to cDC2s [8] (Figure 1).

Like humans, the transcription factor (TF) profile of mouse tDCs formed a continuum between pDCs and cDC2s (Figure 1). TCF4, a TF associated with pDC development [18, 19], was highly expressed in tDC^lo^ (pDC‐like cells) and decreased in tDC^hi^ as cells became more similar to cDC2s [7, 8]. Conversely, *ID2*, which promotes cDC development by antagonizing TCF4 [20], was predominantly expressed by tDC^hi^ in both humans and mice [8]. IRF8, a TF associated with pDC function but not their development [21, 22], was also higher in tDC^lo^ cells in both species [7, 8]. IRF4, a TF associated with cDC2 [23], was highly expressed in tDCs, particularly tDC^hi^ [8]. *Zbtb46*, a TF uniquely expressed in cDCs and required for their function but not their development [24, 25, 26], was expressed by mouse tDCs at intermediate to high levels, increasing from tDC^lo^ to tDC^hi^ [8]. Thus, in both species, tDCs demonstrated a stage‐specific pattern of TF expression, correlating with their transitional phenotype between pDCs and cDC2s. Some of these TFs are essential for tDC development, while others are involved in their differentiation into tDC2s (see Sections 5 and 8).

Morphologically, mouse and human tDCs shared intermediate features that did not align fully with either pDCs or cDCs. Human tDCs exhibited limited cytoplasm, homogeneously distributed mitochondria, a large cerebriform nucleus, and a relatively sparse rough endoplasmic reticulum—features shared with mouse tDCs, rare in pDCs but more prominent in cDCs [6, 10, 12]. More recent analyses revealed a progressive shift in morphology along the tDC^lo^‐to‐tDC^hi^ continuum: tDC^lo^ cells displayed a round morphology and high circularity index, resembling pDCs, whereas tDC^hi^ cells exhibited reduced circularity and increased dendrite formation—morphological traits characteristic of cDCs [27].

Finally, mouse and human tDCs shared similar functional capabilities: the inability to produce IFN‐I in response to viral mimics and a capacity to promote allogeneic T cell proliferation in a mixed leukocyte reaction (MLR)—a function that increased across the tDC^lo^ to tDC^hi^ spectrum and correlated with the expression of MHC‐II/HLA‐DR and co‐stimulatory molecules [6, 8].

Altogether, mouse and human tDCs display a conserved transcriptional and phenotypic continuum, TF profile, and functional features. Similarly, tDCs have recently been observed in the lymphoid organs of nonhuman primates, where their numbers increased rapidly following inoculation with a saponin‐based adjuvant [28]. tDCs have also been identified in swine, suggesting that interspecies alignment extends beyond mice, primates, and humans [29]. Porcine tDCs exhibit a transcriptomic profile intermediate between pDCs and cDC2s, consistent with the transitional identity observed in other species. Notably, porcine tDCs comprise a substantial fraction of circulating DCs and can be subdivided based on their resemblance to either pDCs or cDC2s. In line with human data, CD2 and CD5—two hallmark surface markers of human tDCs—are also expressed on porcine tDCs [29]. These findings further support the evolutionary conservation of this DC population that bridges the pDC and cDC2 lineages.

## 4 A Retrospective Alignment With Previously Identified “Atypical” DCs

Both early and contemporary studies in mice have captured portions of the tDCs spectra (Table 1). One of the earliest descriptions came from the work of Jung and Reizis, who identified what they termed “noncanonical” DCs over a decade ago based on their atypical phenotype and functional profile. In that study, a subset of splenic CD8α^+^ DCs—previously considered a homogeneous cDC1 population—was found to express Cx3cr1 using *Cx3cr1* ^GFP^ mice [16]. Despite expressing CD8α, these cells lacked other hallmark cDC1 features: they did not express canonical cDC1 surface markers such as *Cd205*, *Xcr1* and CD103, lacked expression of the pattern recognition receptor TLR3, failed to secrete IL‐12, and were not dependent on Batf3 for their development. They also diverged from pDCs in several respects, including lacking expression of key markers such as B220 and PDCA‐1 (also known as Bst2 or CD317), and an inability to produce IFN‐I in response to influenza virus stimulation ex vivo. However, like pDCs, noncanonical DCs exhibited immunoglobulin heavy‐chain (IgH) D–J rearrangements—a “lymphoid” feature that reflects the activity of the E protein Tcf4 [18, 19, 20]—suggesting a shared developmental trajectory. Only after the identification of tDCs, and the advent of high‐dimensional transcriptomic and proteomic comparative analyses, was it recognized that noncanonical DCs represent a fraction of tDCs, specifically the CD8α^+^ fraction, which makes up about one‐third of the total population and is enriched within tDC^hi^ [8]. Importantly, most tDCs lack CD8α, underscoring that CD8α is not a reliable marker for defining the tDC population. This clarified that noncanonical DCs are not a separate population but part of the broader tDC continuum.

**TABLE 1:** Comparative nomenclature of tDC and cDC2 subsets across studies.

Nomenclature proposed in Ref. [17]		tDC and tDC2				Canonical cDC2A			cDC2B	
		tDC^lo^	tDC^hi^	CD11b^−^ tDC2	tDC2 (CD11b^+^ Esam^+^ Cx3cr1^+^)	Pre‐cDC2	Early canonical cDC2A	Canonical cDC2A (CD11b^+^ Esam^+^ Cx3cr1^+^)	Pro‐cDC2B	cDC2B
TFs	Tcf4 dependency	[8, 11, 17]								
	Irf4 dependency			[17]						
	Klf4 dependency		[10, 11]			[11, 30, 31]			[11, 31, 32]	
Nomenclature over the years	[16]		Noncanonical DCs (~30% of tDCs)							
	[33]				Esam^+^ cDC2		Cx3cr1^+^ cDC2*	Esam^+^ cDC2		Cx3cr1^+^ cDC2
	[9]	pDC‐like (~60% of tDCs)								
	[34]				cDC2A		cDC2B*	cDC2A		cDC2B
	[35]	Zbtb46^+^ Ly6D^+^ Siglec‐H^+^ stage								
	[10]	pDC‐like	CX3CR1^+^ cDC2b		ESAM^+^ cDC2a		CX3CR1^+^ cDC2b*	ESAM^+^ cDC2a		CX3CR1^+^ cDC2b
	[32]	Pre‐DC2			DC2	Pre‐DC2		DC2	Pro‐DC3	DC3
	[31]	pDC‐like	cDC2b		cDC2a		cDC2b*	cDC2a	Pro‐DC3	DC3
	[36]	Siglec‐H^+^ pre‐cDC2A	CD8^+^ tDCs (~30% of tDCs)		cDC2A	Siglec‐H^−^ pre‐cDC2B	Early cDC2A	cDC2A		cDC2B
	[11]	Pre‐DC2A	DC2A		N/A	Pre‐DC2B	DC2B	N/A	Pro‐DC3	DC3

*Note:* Table summarizes the relationships between the nomenclature used in this review (originally proposed in [17]) and other terms applied to tDCs and cDC2s across studies. Asterisks (*) indicate predictions based on the shared expression of CD11b and Cx3cr1 in early canonical cDC2As and other subsets.

Abbreviation: N/A, not analyzed.

Additional atypical DC populations aligned with tDCs were observed using *Irf8* knockout (KO) mice. These animals lack mature pDCs in the bone marrow and spleen but harbored a population of “pDC‐like” cells expressing intermediate levels of pDC markers such as Siglec‐H and PDCA‐1 [8, 9]. Subsequent studies using *Zbtb46* ^*GFP*^ reporter mice—a TF highly expressed in cDCs but not steady‐state pDCs [24, 26]—further revealed that these pDC‐like cells expressed cDC‐associated features like CD26, depend on Flt3L, and shared pDC‐associated transcriptional regulators, such as *Irf8*, *Runx2*, *SpiB*, and *Tcf4* [10]. However, pDC‐like cells appeared to overlap with only a fraction of tDCs (specifically those labeled in *Zbtb46* ^GPF^ reporter mice, which corresponded to ~60%)—predominantly within tDC^lo^—a finding confirmed by work from Dalod and colleagues [27].

In humans, at least two early studies reported heterogeneity within the pDC compartment likely reflecting tDCs. In one, Palucka and colleagues identified a CD2^high^ pDC subset with reduced IFN‐I production but increased expression of costimulatory molecules and an enhanced capacity to stimulate allogeneic T cells [37]. In another, Engleman and colleagues described a CD2^high^CD5^+^CD81^+^ cells that expressed classical pDC markers but was deficient in IFN‐I secretion, enriched for CD80 expression, and was a potent activator of B cells, antibody production, and regulatory T cell generation [14]. In both cases, cells exhibited features consistent with human tDCs, including AXL expression and functional properties aligning with cDCs, suggesting that these earlier observations likely captured transitional states now recognized as tDCs.

Together, these retrospective analyses reveal that previously described atypical DC populations—such as noncanonical DCs, pDC‐like cells, and functionally altered human pDC subsets—are encompassed within the broader framework of tDCs. This unified classification reconciles long‐standing observations of DC heterogeneity that could not be fully explained by classical pDC, cDC1, or cDC2 definitions, and demonstrates that tDCs are a biologically relevant component of the DC landscape across species.

## 5 tDC Developmental Origins

### 5.1 Shared Ontogeny Between tDCs and pDCs

Evidence that tDCs share developmental features with pDCs accumulated rapidly after their initial identification. pDCs are known to exhibit lymphoid‐associated traits, including immunoglobulin heavy‐chain (IgH) D–J rearrangements and expression of pre‐T cell receptor alpha (PTCRA) [38, 39]. These same features were also detected in tDCs but were absent in most cDCs [8, 16, 17], underscoring the closer developmental alignment of tDCs with pDCs. As noted above, tDCs express the pDC lineage‐defining TF Tcf4, and loss of Tcf4 disrupted the development of both pDCs and tDCs in a cell‐intrinsic manner, as shown using *Cd11c*‐Cre × *Tcf4* ^flox/flox^ mice and mixed bone marrow chimeras [8, 17, 18, 19]. Supporting a similar role in human tDCs, ATAC‐seq analysis revealed chromatin accessibility at TCF4 target sites [15], and patients with Pitt‐Hopkins Syndrome, caused by TCF4 haploinsufficiency, exhibited a downward trend in tDC abundance (gated as pre‐cDCs [12]), although this trend has yet to reach statistical significance due to small sample size (*n* = 4 donors).

In addition to Tcf4, mouse tDCs express high amounts of Irf8, *Bcl11A*, *Runx2*, and *SpiB* [8]—TFs implicated in pDC development that are positioned upstream or downstream of Tcf4 [18, 40, 41]. *IRF8, RUNX2*, and *BCL11A* have also been shown to be active in human tDCs based on chromatin accessibility profiling [15, 42]. Like pDCs, tDCs (gated as noncanonical DCs) require Mtg16 (also known as Cbfa2t3), a transcriptional cofactor of the ETO protein family [43], the histone deacetylase HDAC1 [44], and the transcriptional repressor Trim33 [45, 46] for their development.

The developmental alignment between tDCs and pDCs was further reinforced by lineage tracing. Two independent pDC‐specific models—*hCD2*‐Cre × *Rosa* ^LSL‐EYFP^, which relies on lymphoid features, and *Cd300c* ^Cre^ × *Rosa* ^LSL‐tdTomato^, which labels pDCs independently of such features—showed that ~80% of tDCs were labeled, whereas labeling was minimal in cDCs, indicating a shared ontogeny with pDCs [10, 17]. Importantly, adoptive transfer of pDCs did not give rise to tDCs, and selective depletion of pDCs did not affect tDC numbers at steady state, demonstrating that tDCs do not arise from pDCs under homeostatic conditions [17]. Instead, both tDCs and pDCs appear to derive from a shared upstream progenitor.

All DCs arise from a Cx3cr1‐expressing hematopoietic progenitor population in the bone marrow through a Flt3L‐dependent pathway [47, 48, 49, 50, 51]. cDC primarily derive from CD115‐expressing conventional DC progenitors (pro‐cDC) [52, 53], with cDC1 and cDC2 originating from Siglec‐H^−^ Ly6C^−^ and Siglec‐H^−^ Ly6C^+^ immediate precursors, respectively [54, 55]. Although some pDCs can arise from lymphoid progenitors [9, 56, 57], the majority originate from a pDC progenitor (pro‐pDC) that expresses CD115^−^ Cx3cr1^+^ Ly6D^+^ Siglec‐H^+^ [47, 58, 59]. Flt3L‐supplemented bone marrow cultures recapitulate these pathways in vitro and, importantly, give rise to tDCs [17], confirming their bone marrow origin.

**FIGURE 2:**
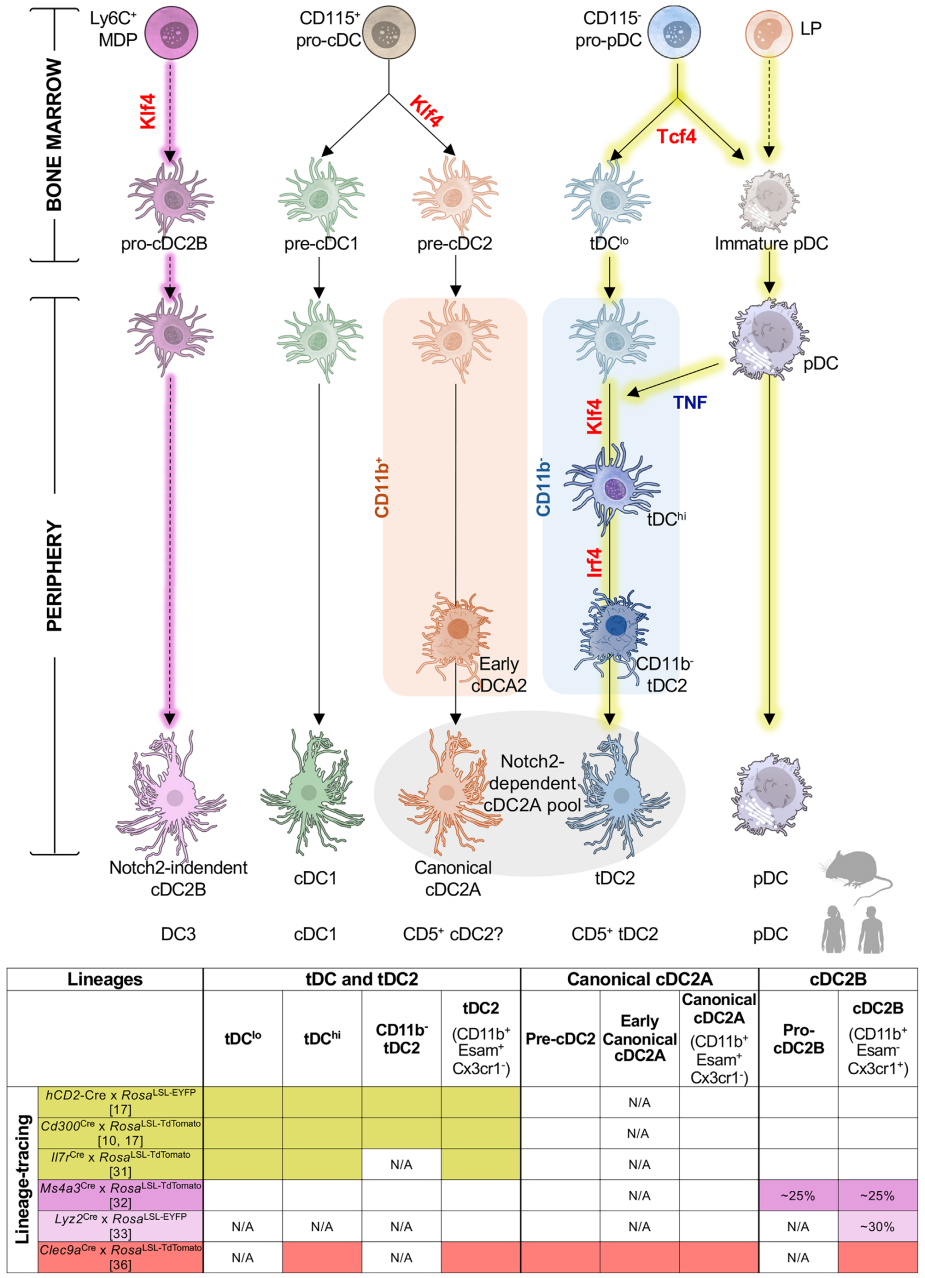
Diagram of the DC network incorporating tDC development. Schematic representation of the major developmental pathways leading to the different DC lineages in mice, including the tDC branch. cDC1s and canonical cDC2As arise from CD115^+^ pro‐cDCs via committed pre‐cDC1 and pre‐cDC2 intermediates. pDCs develop from CD115^−^ pro‐pDCs, which also give rise to tDCs. The tDC branch follows a unidirectional differentiation trajectory from tDC^lo^ → tDC^hi^ → CD11b^−^ tDC2 → tDC2s (CD11b^+^ Esam^+^ Cx3cr1^−^). This pathway runs in parallel to the canonical pre‐cDC2 differentiation route, which proceeds via an early cDC2A intermediate before maturing into canonical cDC2As (CD11b^+^ Esam^+^ Cx3cr1^−^). tDCs may also originate from pDCs under conditions of high TNF and low IFN‐I. Solid arrows indicate established developmental relationships, whereas dashed arrows denotate unresolved or controversial steps. The diagram also highlights key TFs (labeled in red) required at major branch points. MDP, monocyte‐dendritic cell progenitor; pro‐cDC, cDC progenitor; pro‐pDC, pDC progenitor; LP, lymphoid progenitor. The accompanying table summarizes lineage relationships supported by lineage‐tracing models, with “N/A” indicating populations not analyzed. Elements of this figure were created with resources from the NIH BioArt Source.

To identify the bone marrow progenitor of tDCs, single‐cell RNA sequencing and CyTOF analyses of CD135^+^ bone marrow progenitors were performed [17]. This approach inferred a developmental trajectory from CD115^−^ pro‐pDCs to tDCs, further reinforcing their alignment with the pDC lineage. In support of this, pro‐pDCs, like pDCs and tDCs, exhibited high (~80%) EYFP labeling in *hCD2*‐Cre × *Rosa* ^LSL‐EYFP^ mice and were depleted in *hCD2*‐Cre × *Cx3cr1* ^LSL‐DTR^ mice [17, 47]. To directly test pro‐pDC ability to differentiate into tDCs and pDCs, adoptive transfer experiments were performed. Purified pro‐pDCs gave rise to both pDCs and tDCs when transferred into nonirradiated congenic mice, establishing a shared developmental origin (Figure 2) [17]. Notably, a small portion of cDC2‐like cells also emerged in these experiments—cells that, in retrospect, correspond to tDC2s (see Section 8).

Pro‐pDCs express surface markers similar to those reported for the bone marrow progenitor described by Dr. Krug and colleagues, which gave rise to pDCs, an intermediate population overlapping with tDCs, and cDC2‐like cells [35]. Our pro‐pDC gate also includes CD115^−^ CD127^−^ Ly6D^+^ Siglec‐H^+^ cells, previously identified by Dr. Tussiwand and colleagues as precursors of pDCs and cDC2‐like cells, and shown in our hands to also give rise to tDCs [9, 17]. Altogether, these studies support the capacity of CD115^−^ pro‐pDCs to generate pDCs, tDCs and cDC2‐like cells—likely representing tDC2s. Importantly, our pro‐pDCs gate encompasses Siglec‐H^+^ Ly6C^+^ cells recently suggested to give rise to tDCs (gated as pre‐cDC2A) and cDC2‐like cells (gated as cDC2A) rather than pDCs [11, 36], suggesting that pro‐pDC may represent a heterogeneous mixture of fate‐restricted precursors that requires further analysis.

Crucially, adoptive transfer of pro‐pDC revealed a temporal sequence of tDC development: tDC^lo^ cells appeared early (day 2), while tDC^hi^ cells emerged later (day 4), indicating a stepwise progression from tDC^lo^ to tDC^hi^ (Figure 2) [17]. These findings were the first to link the two subpopulations, demonstrating that tDC^lo^ and tDC^hi^ represent a continuum of differentiation states arising sequentially from the same progenitor pool [17]. This model is supported by several lines of evidence. First, both tDC^lo^ and tDC^hi^ share pDC‐like developmental features, including IgH rearrangements and PTCRA expression [8]. Second, both subpopulations intrinsically require Tcf4 for their development [17]. Third, both are labeled at comparable frequencies (~80%) in lineage‐tracing models [17]. Fourth, both display a distinct transcriptomic signature that sets them apart from other DCs [17, 27]. Finally, adoptive transfer of tDC^lo^ gives rise to tDC^hi^, directly demonstrating their developmental linkage [17]. As tDC^lo^ transition to tDC^hi^, they upregulate TFs associated with cDCs, including Zbtb46, Klf4 and Irf4 [8, 10]. This upregulation reflects a transcriptional reprogramming process that culminates in differentiation into tDC2s, rather than indicating different origins for tDC^lo^ and tDC^hi^ [9], or a developmental alignment of tDCs with cDCs instead of the pDC lineage [11].

### 5.2 Alternative Developmental Routes in Inflammation

Altogether, our data and those of others demonstrate that tDCs predominantly arise from bone marrow CD115^−^ pro‐pDCs under homeostatic conditions. However, whether this developmental route is the main source during inflammation remains unclear, as tDCs may follow alternative differentiation routes in activated settings. Indeed, our most recent work reveals that human pDCs can undergo functional reprogramming into cDC2‐like cells via a tDC‐like intermediate state—a process driven by TNF and fully inhibited by type I interferon [42]. This finding suggests that inflammatory cues can unlock latent plasticity within pDCs, allowing them to acquire new transcriptional programs and effector functions.

Whether pDC‐derived tDCs differ functionally from pro‐pDC‐derived tDCs remains unknown, but the distinction may have important immunological consequences. Future studies should evaluate the relative contribution of each route under different inflammatory contexts—and determine whether these influence the quality or nature of immune responses established.

## 6 tDC Tissue Distribution

One of the biggest limitations in the analysis of tDC tissue distribution is the lack of specific surface markers or reporter models that allow for their unambiguous identification. Nevertheless, multidimensional analyses integrating multiple markers simultaneously have enabled the resolution and tracking of tDCs across a range of lymphoid and nonlymphoid tissues in both mice and humans. While their frequency is generally low—and comparable to that of cDC1s in several instances—their consistent presence suggests a conserved and potentially specialized functional role.

Mouse tDCs originate in the bone marrow but are rare there [10, 17]. This pattern holds true in humans, where tDCs are ~20‐fold less abundant in the bone marrow compared to other DCs [60]. After their generation from bone marrow progenitors, tDCs enter the bloodstream, where they are detectable in both mouse and human [6, 7], including cord blood, indicating that they circulate from the earliest neonatal stages [61]. In vivo deuterium labeling in humans revealed a rapid appearance of tDCs in the blood—within 6 h of labeling—and a short circulating half‐life (~2.16 days), similar to other cDC subsets [60]. These findings suggest that tDCs spend only a brief period in the bone marrow before entering circulation and subsequently migrating to tissues.

Their presence in other primary lymphoid organs—namely, the thymus—has been described in mice, where tDCs appear around day 21 postnatal [62, 63]. These cells localize near endothelial microvessels and rapidly bind intravenously injected antibodies, suggesting they have direct vascular access and may participate in blood‐borne antigen delivery [63]. Accordingly, thymic tDCs may correspond to previously described pDC‐like thymic DCs that shuttle blood‐borne antigens to the thymus for tolerance induction [64]. However, their exact function within the thymus remains unclear. Whether tDCs are present in the human thymus has yet to be determined.

Secondary lymphoid organs are the best‐characterized sites of tDC residence in both species. In mice, tDCs are consistently detected in the spleen and lymph nodes, comprising approximately 3% of total DCs [8, 9, 10, 17, 27, 62]. In humans, tDCs are also present in lymphoid tissues such as the spleen and tonsils [6, 7, 8]. Nevertheless, their precise microanatomical localization within these organs—whether in B cell follicles, T cell zones, or marginal zones—remains to be determined but will be critical for elucidating their role in antigen presentation and immune modulation.

In peripheral nonlymphoid tissues, tDCs show variable distribution across organs and species at steady state. In mice, tDCs have been detected in the lungs [8, 62], liver [10, 17, 62], and kidneys [10]. Notably, the heart harbors a large tDC population, constituting up to 80% of total cardiac DCs [10]—a striking observation that remains functionally unexplored. In contrast, tDCs are absent from steady‐state skin in both mice and humans [7, 8, 60, 65]. Whether their progeny—tDC2s—are present or absent in these tissues remains unclear and has not been systematically evaluated.

Under inflammatory conditions, tDCs are rapidly recruited to peripheral sites. In mice, their numbers increase significantly in the lung and liver following infection with influenza virus (PR8) and mouse coronavirus (M‐CoV; MHV‐A59) [8, 17]. Similarly, in humans, tDCs are recruited into inflamed skin in a model of cutaneous immune activation [60] and into sterile skin blisters, where they upregulate HLA‐DR, costimulatory molecules and CCR7, especially following house dust mite challenge [65]. tDCs have also been found in the cerebrospinal fluid (CSF) of patients with inflammatory demyelinating diseases (IDD) of the central nervous system (CNS), alongside T and B cells [66]. The same study identified tDCs in the brain tissue of a patient with multiple sclerosis (MS), the most common IDD. Using cell–cell interaction inference tools, tDCs were predicted to engage in homotypic interactions and communicate with other immune cells—including B cells, T cells, and activated DCs—forming a stimulatory network that may prime autoreactive lymphocytes during IDD pathogenesis [66]. The molecular signals that govern tDC recruitment remain undefined.

In summary, the anatomical distribution of tDCs in both mice and humans reveals a conserved but context‐sensitive pattern. Their selective presence in lymphoid organs and inducible recruitment to peripheral tissues suggests roles in both local immune surveillance and central immune coordination.

## 7 tDC Function

Despite their capacity to differentiate into tDC2s, tDCs do not appear to be merely precursors—that is, not just passive intermediates in a differentiation pathway—but are instead functionally active cells with distinct immune roles of their own. They display a unique, conserved, and dynamic effector profile that defines their contribution to innate immune responses and inflammation, and their role in bridging innate and adaptive immunity.

### 7.1 Pathogen Recognition Receptors and Viral Sensing

Mouse tDCs express and respond to a broad array of Toll‐like receptor (TLR) agonists, including TLR1/2, 5, 6, and prominently express TLR7 and 9, highlighting their role as sensors of microbial pathogen‐associated molecular patterns (PAMPs) [10, 17]. In humans, circulating tDCs primarily express TLR7 and 9, with lower expression of TLR1, 6, and 10 at the transcriptional level [6, 12]. These expression patterns resemble those of pDCs and suggest that tDCs, like their developmental relatives, are poised to respond to viral nucleic acids and may play important roles in antiviral immunity.

Unlike pDCs, which preferentially sense viruses through infected cells via the generation of interferogenic synapses [67], human tDCs preferentially capture free viral particles through specific surface receptors. For example, human tDCs express high levels of SIGLEC‐1 (CD169) [6], a surface lectin that facilitates viral capture and transmission of SARS‐CoV‐2, Ebola, and HIV‐1 [68, 69, 70, 71, 72]. SIGLEC‐1 enables both cytosolic and endosomal sensing of HIV‐1 by tDCs—a dual sensing capability unique to tDCs and absent in other DC subsets such as pDCs, cDC1s, and cDC2s [68, 71, 73]. Importantly, SIGLEC‐1 expression has been leveraged for immunotherapy: ganglioside‐containing liposomes targeting SIGLEC‐1 on tDCs have been used in a nanovaccine platform incorporating the TLR7/8 agonist R848 and tumor antigen payloads. This approach triggered cytokine secretion and efficient cross‐presentation to antigen‐specific CD8^+^ T cells [74]. In addition to SIGLEC‐1, human tDCs express AXL, a TAM family receptor exploited by viruses such as Zika and Ebola for host entry [75, 76]. However, the physiological relevance of AXL on tDCs during viral infection remains unclear and warrants further study. These findings collectively highlight a specialized viral‐sensing profile for human tDCs.

In mice, tDCs also appear to directly capture viral particles. Unlike pDCs, mouse tDCs are permissive to infection with Vesicular Stomatitis Virus (VSV) and M‐CoV [77], suggesting distinct antiviral capabilities. However, the specific receptors mediating viral uptake may differ from those in humans: mouse tDCs express low levels of Siglec1 and do not express significant Axl at steady state [8]. Axl may be induced following IFN‐I exposure [78], but it is unclear if IFN‐I modulates the expression of other receptors. These differences likely reflect species‐specific evolution of pathogen recognition mechanisms and underscore the importance of studying the function of tDCs within the context of host‐adapted pathogens.

### 7.2 Activation and Secretory Functions: IL‐1β as a Hallmark of tDC‐Mediated Inflammation

Following pathogen recognition—mimicked by TLR stimulation—tDCs reprogram their transcriptome within 3 h, without immediate differentiation into tDC2s [17], highlighting their distinct immunological role beyond their differentiation output. Interestingly, tDC^lo^ and tDC^hi^ follow distinct activation trajectories. For example, during murine cytomegalovirus (MCMV) infection, tDC^lo^ cluster with pDCs, whereas tDC^hi^ cluster more closely with cDC2s and upregulate genes associated with T cell interactions (*Ccr7, Il15, Il15ra, Cd80, Cd200* and *Cd274*) [27]. Accordingly, in vivo stimulation with the TLR9 agonist CpG‐A induces CD69 upregulation in tDC^lo^, mimicking the pDC response, whereas tDC^hi^ upregulate MHC‐II and CD86, mirroring cDC activation [8]. A comparable response pattern is observed in human tDCs following yellow fever vaccination, a live attenuated virus: circulating tDCs maintain their transcriptional identity postvaccination, including expression of AXL, SIGLEC6, and ADAM33 [79]. However, RNA velocity analysis suggests a bidirectional transcriptional response toward both pDCs and cDC2s, again implying functional divergence between tDC^lo^ and tDC^hi^ subpopulations. Whether these divergent activation states are hardwired or environmentally induced remains to be determined. Nevertheless, these findings reinforce that tDCs are not passive precursors, but active responders capable of initiating distinct maturation programs.

Upon sensing pathogens, tDCs produce a wide array of pro‐inflammatory cytokines. Human tDCs secrete IL‐8 in response to CpG‐C and LPS (TLR9 and TLR4 agonists) [6], and IL‐6, TNF and MIP1α following stimulation with R848/LPS/Poly(I:C) (TLR7/8, TLR4, and TLR3 agonists) [6, 12]. Similarly, the TLR9 agonist CpG‐C induces higher levels of IL‐8, IL‐12p40, and IL‐6 in human tDCs than in pDCs [14]. Importantly, unlike pDCs—which are well‐established as potent IFN‐I producers—tDCs generally do not appear to secrete detectable levels of IFN‐I, at least under the experimental conditions tested to date, in both mouse and human systems [6, 8, 9, 14, 15, 16, 77]. A recent report challenged this view, suggesting that tDCs produced more IFN‐I than pDCs [11]; however, this finding contrasts with previous reports using the same stimuli [9], and a body of evidence shows the specialized IFN‐I secretion of pDCs. Such discrepancies may reflect technical factors—including the gating strategies used to define pDCs and the relative abundance of pDCs in culture—particularly given the described requirement for quorum sensing in pDC‐mediated IFN‐I responses [80]. Nevertheless, the prevailing consensus across multiple studies in both human and mouse models supports the notion that tDCs have a reduced intrinsic capacity to secrete IFN‐I. This functional distinction correlates with their lower expression of IRF7 [8], a TF critical for robust IFN‐I production in pDCs [81, 82].

A defining feature of tDCs is their ability to produce high levels of interleukin‐1 beta (IL‐1β)—a central pro‐inflammatory cytokine. Both mouse and human tDCs secrete markedly more IL‐1β than pDCs or cDCs following sensing CpG‐C, influenza virus, human cytomegalovirus (CMV), or M‐CoV [6, 17, 71]. tDCs may also respond to bacterial products by secreting IL‐1β: in a human skin blister model, tDCs upregulated *IL1B* transcripts following intradermal injection of UV‐killed *E. coli* [60]. These findings suggest IL‐1β secretion by tDCs may extend beyond antiviral responses to broader roles in microbe‐induced inflammation. However, it remains unclear whether both tDC^lo^ and tDC^hi^ subpopulations contribute equally to this function. Notably, only splenic tDCs—but not their tDC2 progeny—produce IL‐1β [17], indicating that this function is lost upon differentiation. The mechanisms underlying IL‐1β secretion in tDCs remain undefined, and whether they involve canonical inflammasome activation or an alternative pathway is an open question.

Tight regulation of IL‐1β secretion is essential to avoid immunopathology [83, 84]. Recent evidence suggests that pDCs may play a regulatory role by restraining the secretion of IL‐1β by tDCs [17]. Indeed, during M‐CoV infection, pDC depletion results in excessive IL‐1β production by tDCs and severe immunopathology. This mirrors human data from severe viral infections, such as COVID‐19, where IFN‐I deficiency correlates with hyperinflammation, and IL‐1β blockade has shown therapeutic benefit [85, 86, 87]. Whether IFN‐I directly restrains tDC activation or acts through other intermediaries remains unknown.

### 7.3 Antigen Presentation, T Cell Priming and Polarization: Functional Parallels With cDCs

Antigen capture represents the first step in the initiation of adaptive immune responses by DCs. While a comprehensive comparison between tDCs and other DC subsets is still lacking, tDCs appear to be highly efficient at antigen uptake. In mice, tDCs—especially tDC^hi^—internalize both soluble (e.g., ovalbumin) and particulate (e.g., sheep red blood cells) antigens in vivo and ex vivo, and they do so more efficiently than pDCs and at levels comparable to cDC2s [17].

Following antigen recognition, tDCs demonstrate a strong capacity to activate the proliferation of CD4^+^ T cells as shown in mixed leukocyte reactions (MLRs) of both human and murine models [6, 7, 8, 10, 14, 37], and antigen‐specific T cell responses in murine models [10, 17]. T cell activation is significantly enhanced in tDC^hi^ compared to tDC^lo^, consistent with higher expression of MHC‐II and costimulatory molecules in the former [17]. Still, both subpopulations outperform pDCs, and tDC^hi^ is comparable—or even superior—to cDC2s in their capacity to activate naïve T cells [17].

Evidence for cross‐presentation to CD8^+^ T cells is more limited. In humans, purified tDC^lo^ and tDC^hi^ from blood can promote allogeneic CD8^+^ T cell proliferation in vitro following stimulation with the TLR4 agonist LPS and the TLR7/8 agonist R848 [6]. However, this likely reflects increased MHC‐I expression and costimulatory activity rather than bona fide cross‐presentation. Nevertheless, the magnitude of CD8^+^ T cell proliferation was comparable to that induced by cDC1s and cDC2s [6]. In contrast, cross‐presentation does not appear to be a feature of mouse tDCs. An early study showed that tDCs (gated as noncanonical DCs) are not depleted following cytochrome c inoculation [16]—a classical method to eliminate cross‐presenting cells [88]—suggesting an inability to cross‐present. Similarly, mouse tDCs (gated as pDC‐like cells) do not cross‐present ovalbumin (OVA)‐loaded apoptotic splenocytes to OVA‐specific CD8^+^ T cells as efficiently as cDC1s [10].

Very little is known about the ability of tDC to influence T cell polarization. In humans, it has been suggested that tDCs can polarize allogeneic naïve CD4^+^ T cells toward Th2, Th9, Th22, Th17 [71], and Treg [14] fates but are relatively poor inducers of Th1 responses [71]. However, systematic analyses using antigen‐specific models are lacking. In mice, some evidence points to a preferential role for tDCs in promoting Th17 differentiation: when stimulated with *C. albicans* , Pam3CSK4, and R848, they induce higher IL‐17 secretion by naïve CD4^+^ T cells—exceeding the levels induced by pDCs or cDCs [10]. This trend also appears in vivo: in *Cd11c*‐Cre × *Klf4* ^fl/fl^ conditional knockout (cKO) mice—where Klf4 is necessary for tDC‐to‐tDC2 conversion—there is a reduction in IL‐17‐producing CD4^+^ T cells in the skin and draining lymph nodes at steady state [10]. However, earlier work showed that Klf4‐expressing migratory DCs from skin‐draining lymph nodes preferentially promote Th2, rather than Th17, responses [30]. This apparent discrepancy may reflect the fact that *Klf4* cKO mice have defects in several DC subsets—including tDCs, tDC2s, cDC2As, and cDC2Bs (see Section 9) [10, 11, 30]—making it difficult to attribute functional outcomes to a single subset.

Regarding the crosstalk between tDCs and B cells, an early study in humans showed that CpG‐B‐stimulated tDCs promote B cell activation and antibody production in vitro [14]. Blocking CD70—the ligand for CD27—on tDCs reduced antibody secretion, suggesting that CD70–CD27 signaling plays a key role in tDC–B cell crosstalk. Further research is needed to determine whether this function is conserved in mice.

Altogether, these findings show that tDCs combine broad innate sensing and robust IL‐1β secretion with potent T cell priming and potential involvement in T cell polarization and B cell support. While many of these functions overlap with those of cDCs, some appear unique to tDCs, such as IL‐1β production. Future work should aim to clarify the immunological settings in which tDCs and tDC2s dominate specific outcomes, and whether their roles are complementary, redundant, or specialized relative to other DC subsets. Dissecting these roles will be critical for understanding their therapeutic potential in infection, autoimmunity, and vaccination.

## 8 The tDC‐to‐tDC2 Differentiation Pathway

Before discussing the differentiation trajectory of tDCs, it is important to clarify the nomenclature used in this section, given the current lack of consensus in the field (see Table 2). Historically, the prefix “c” in cDC2s refers to “conventional”, based on the assumption that these cells arise exclusively from CD115^+^ conventional progenitors, distinct from pDCs. However, it is now clear that this population includes cells of distinct developmental origins, including those derived from tDCs. To avoid further confusion and maintain consistency with the literature, we retain the term cDC2s here to collectively refer to CD11c^hi^ CD11b^+^ MHCII^+^ CD172a^+^ cells, regardless of their origin. As discussed in Section 9, growing evidence supports the presence of distinct subpopulations within this gate. The primary division in the mouse spleen lies between Notch2‐dependent Esam^+^ Cx3cr1^−^ cDC2s (referred to as cDC2A) and Notch2‐independent Esam^−^ Cx3cr1^+^ cDC2s (referred to as cDC2B). Importantly, as discussed below, the cDC2A pool itself is not developmentally uniform: it comprises two lineages with distinct origins. One branch arises from tDCs and will be referred to here as tDC2s. The other arises from canonical pre‐cDC2 precursors and will be referred to as canonical cDC2As (Figure 2 and Table 2).

**TABLE 2:** Dendritic cell subset definitions used in this manuscript.

Subset	Key markers/Description	Notes
cDC1	CD11c^hi^ CD11b^−^ MHCII^+^ Xcr1^+^	
cDC2	CD11c^hi^ CD11b^+^ MHCII^+^ CD172a^+^	Encompasses cDC2A and cDC2B populations
1. cDC2A	Esam^+^ Cx3cr1^−^	Notch2‐dependent. Encompasses tDC2s and canonical cDC2As
(a) tDC2	Esam^+^ Cx3cr1^−^; traced to pro‐pDC/pDC/tDC (e.g., labeled in *hCD2*‐Cre x*Rosa* ^LSL‐EYFP^)	tDC‐derived cells
(b) Canonical cDC2A	Esam^+^ Cx3cr1^−^; not traced to pro‐pDC/pDC/tDC (e.g., not labeled in *hCD2*‐Cre x*Rosa* ^LSL‐EYFP^)	Pre‐cDC2‐derived cells
2. cDC2B	Esam^−^ Cx3cr1^+^	Notch2‐independent.

*Note:* This table summarizes how dendritic cell subsets are defined throughout the manuscript, including key phenotypic features and their developmental relationships. tDC2s and canonical cDC2As share an identical surface phenotype in mouse spleen (i.e., expression of core cDC2 markers plus Esam^+^ Cx3cr1^−^) and can only be distinguished by lineage history: tDC2s trace to the pro‐pDC/pDC/tDC lineage (e.g., *hCD2*‐Cre *× Rosa* ^LSL‐EYFP^), whereas canonical cDC2As are not labeled in these lineage‐tracing models and instead arise from CD115^+^ pre‐cDC2 precursors.

### 8.1 tDC Differentiation Into tDC2s

The phenotypic continuum of tDCs—from tDC^lo^ cells to tDC^hi^ cells—together with the appearance of cDC2‐like cells following pro‐pDC and tDC adoptive transfers, supports a differentiation trajectory from tDCs to cDC2‐like cells. This was first explored in humans: Villani et al. demonstrated that human tDCs (gated as ASDCs) can differentiate into CD1c/BDCA1^+^ cells, but not CLEC9A^+^ cDC1s [6]. See et al. reported similar observations, although they found a small fraction (< 2.5%) of cells acquiring cDC1 identity [12]. This rare output was not reproduced by subsequent studies and likely reflects minor contamination with other progenitor populations in the initial cell preparations. More recently, stringent purification strategies and CyTOF‐based analyses confirmed that human tDCs differentiate exclusively into cDC2‐like cells and not cDC1s [17].

In mice, lineage‐tracing approaches support the same conclusion: tDCs can differentiate into cDC2‐like cells but not efficiently into cDC1s [10, 17]. These experiments further revealed that around 25% of all splenic cDC2s trace back to pro‐pDCs/pDCs/tDCs [10, 17], underscoring their limited contribution to the overall splenic cDC2 compartment. Indeed, selective ablation of pro‐pDCs/pDCs/tDCs reduced only the fraction of cDC2s lineage‐traced to them, without affecting the rest [17]. Moreover, only the fraction that traces to pro‐pDCs/pDCs/tDCs exhibits immunoglobulin heavy‐chain (IgH) rearrangements, reinforcing their developmental connection [17]. Direct functional validation came from adoptive transfer experiments [10, 17]: tDCs—but not pDCs—transferred into nonirradiated congenic mice gave rise to cDC2‐like cells at steady state. At early time points (Day 2), these cells expressed several cDC2 markers, albeit at lower levels, and crucially, lacked CD11b expression; these cells were therefore named CD11b^−^ tDC2s [17]. By Day 4, they acquired the full cDC2 phenotype, including expression of CD11b [17]. This fully differentiated progeny is referred to as tDC2s, defined as the tDC‐derived population that has all the features of fully differentiated cDC2s, i.e., CD11c^hi^ CD11b^+^ MHCII^+^ CD172a^+^.

Notably, adoptive transfer experiments demonstrated that tDC^lo^ give rise to tDC^hi^, CD11b^−^ tDC2s and tDC2s; tDC^hi^ generate only CD11b^−^ tDC2s and tDC2s; and CD11b^−^ tDC2s progress exclusively to tDC2s. These results support a stepwise and unidirectional progression of differentiation: tDC^lo^ → tDC^hi^ → CD11b^−^ tDC2 → CD11b^+^ Esam^+^ tDC2 (Figure 2) [17]. This trajectory may have been overlooked in studies that did not include CD11b as a core cDC2‐defining marker [10]. Importantly, in our hands, the final step consistently yields CD11b^+^ Esam^+^ Cx3cr1^−^ tDC2s—demonstrating that tDCs differentiate into cDC2A‐like cells, but not cDC2B [17].

A similar pattern emerges in humans: tDCs generate CD5^+^ CD14^−^ cDC2s, consistent with a cDC2A‐like cell identity and distinct from cDC2B (usually called DC3 in humans), which typically express CD14 (see Section 9). Importantly, under all tested conditions—in both mouse and human systems—tDCs did not give rise to pDCs or cDC1s, arguing strongly against their classification as multipotent progenitors. Instead, these findings highlight a predominantly lineage‐restricted and unidirectional commitment to a subpopulation of cDC2s.

### 8.2 Divergent Origins of Notch2‐Dependent cDC2A: tDCs Vs. Canonical Pre‐cDC2s

Although tDCs can differentiate into tDC2s, these cells account for only ~35%–40% of the splenic cDC2A pool expressing CD11b^+^ Esam^+^ Cx3cr1^−^ [17]. The majority instead derives from Siglec‐H^−^ Ly6C^+^ pre‐cDC2s, the canonical cDC2 precursor described by the groups of Murphy and Ginhoux that originates from a CD115^+^ cDC progenitor (pro‐cDC) [54, 55] (Figure 2). To distinguish pre‐cDC2‐derived progeny from tDC2s, we refer to these cells here as canonical cDC2A (see Table 2).

Despite original suggestions that tDCs—particularly tDC^lo^—correspond to original definitions of canonical pre‐cDC2s [10], several lines of evidence highlight a clear developmental distinction between the two [17]. First, pre‐cDC2s do not share ontogeny with pDCs; therefore, these cells are not labeled in pro‐pDC/pDC/tDC lineage‐tracing mouse models [10, 17] and remain present in pro‐pDC/pDC/tDC lineage‐depletion models [17]. Second, although canonical pre‐cDC2s and tDCs share expression of certain markers such as Cx3cr1, multidimensional clustering analyses consistently separate them into distinct phenotypic spaces [17]. These differences are reflected in the expression of TFs and surface proteins, including Tcf4, Irf8, Siglec‐H, and CD8. Finally, adoptive transfer experiments show that both canonical pre‐cDC2s and tDCs differentiate into cells classified as Notch2‐dependent cDC2A (CD11b^+^ Esam^+^ Cx3cr1^−^); however, only tDC2s—not canonical cDC2A—retain the pro‐pDC/pDC/tDC lineage‐tracing label and IgH rearrangements [17], confirming that tDCs and their progeny represent a distinct developmental origin.

Functionally, tDCs and canonical pre‐cDC2s also diverge [17]. Pre‐cDC2s exhibit higher proliferative capacity and turnover [11, 17]. In contrast, tDCs are more quiescent yet functionally poised, showing superior capacity for antigen uptake, processing, and presentation, as well as robust cytokine secretion following stimulation (see Section 7). Differentiation trajectories further distinguish them: tDCs transition through a CD11b^−^ tDC2 intermediate stage [17], whereas canonical pre‐cDC2s progress through a CD11b^+^ early cDC2A stage [36]. Moreover, tDCs are rare in the bone marrow, suggesting rapid exit after development, while pre‐cDC2s are more abundant in that niche [17]. Taken together, these findings highlight two independent developmental lineages contributing to the cDC2A pool (Figure 2) [17].

These two routes of cDC2A development—via tDCs and canonical pre‐cDC2s—appear to be governed by divergent transcriptional programs (Table 1). For instance, Tcf4 is essential for tDC development [8, 11, 17], and tDC2 numbers are predictably reduced in its absence, although this has not yet been directly tested. Irf4, a well‐established TF associated with cDC2s, is highly expressed in tDC^hi^ and appears necessary for the tDC^hi^‐to‐CD11b^−^ tDC2 transition, but not for the canonical pre‐cDC2‐to‐cDC2A differentiation [17]. This aligns with findings in *Irf4* cKO mice that show reduced numbers of cDC2s expressing CD24 [89, 90, 91, 92]—a marker moderately enriched in tDC2s [17]. Conversely, the TF Klf4 regulates the development of canonical pre‐cDCs but not tDCs [10, 30], a finding recently confirmed by others [11]. However, Klf4 is required for the tDC^lo^‐to‐tDC^hi^ transition [10, 11], which may explain the absence of CD11b^−^ cDC2s in the skin draining lymph nodes of *Klf4* cKO mice [30]—a DC subset that may correspond to tDC‐derived cells based on their phenotype. Irf8, critical for cDC1 development, is dispensable for tDC and pDC development [8, 21]. However, its absence leads to accumulation of aberrant pDC and tDC (pDC‐like) populations with potentially altered function [8, 9, 21]. The role of Irf8 in tDC‐to‐tDC2 differentiation remains unresolved. Additional regulators—including Rbpj‐Notch2, which influences the final differentiation step of splenic cDC2A [33, 36, 93, 94], and Zeb2, which controls cDC and pDC development across tissues [95, 96]—warrant systematic evaluation with lineage‐tracing and adoptive transfer models.

### 8.3 Converging Outputs: tDC2s and Canonical cDC2As as Similar Products

Despite their distinct developmental origins, tDC2s and canonical cDC2As share nearly identical gene expression profiles at steady state. Bulk RNA sequencing—which provides greater depth than single‐cell approaches—showed that these two populations are largely transcriptionally indistinguishable in the spleen ([17]; unpublished observations). At the protein level, however, subtle differences remain: when identified by lineage tracing, tDC2s express slightly lower levels of CD11b and higher levels of CD24, CD45RB and CD45RA compared to their canonical cDC2A counterparts [17]. Yet, these modest differences are insufficient to reliably distinguish the two populations without lineage‐tracing models, underscoring the challenge of phenotypically resolving these splenic populations.

Genetic models further support that two independent developmental sources contribute to the cDC2A pool. In mice with impaired tDC development (e.g., Tcf4 deficiency), or when tDCs are depleted, the cDC2A pool number is preserved [11, 17], suggesting that canonical pre‐cDC2s can compensate for the loss of tDC‐derived input. Conversely, when canonical pre‐cDC2s are ablated (e.g., *Clec9a*‐Cre x *Rosa* ^LSL‐DTA^ models), the cDC2A pool remains intact, but the resulting cells carry immunoglobulin heavy‐chain (IgH) rearrangements typical of the pro‐pDC/pDC/tDC lineage [97]—pointing to a compensatory contribution from tDCs. This reciprocal compensation suggests a dynamic equilibrium between the two pathways, with either source able to sustain the compartment under stress or perturbation. The convergence of these dual inputs into the CD11b^+^ Esam^+^ Cx3cr1^−^ cDC2A pool is likely shaped by tissue‐derived signals such as Notch2, lymphotoxin, and retinoic acid (Figure 2) [33, 36, 93, 94, 98, 99, 100, 101].

Although convergence produces a unified population, it remains unresolved whether tDC2s and canonical cDC2As retain functional imprints of their distinct origins or instead represent a single terminal differentiation state with overlapping functions. If the latter is true, then the functional uniqueness may reside in the developmental intermediates—particularly in tDCs, which display distinctive effector capabilities prior to differentiation (see Section 7). Why evolution has maintained dual inputs into the cDC2A pool remains unclear. One possibility is simple developmental redundancy, ensuring that the compartment can be sustained even if one pathway is impaired. Alternatively, different tissues may preferentially recruit one subset over the other in response to local cues, or immune challenges may selectively engage one developmental route depending on the context. Future studies will be essential to define the relative contributions of each pathway across tissues, immune contexts, aging, and disease.

## 9 Situating tDCs and tDC2s Within the Expanding cDC2 Landscape

The heterogeneity of the cDC2 compartment has long been a topic of debate. Integrating tDCs and their progeny—tDC2s—into the evolving cDC2 classification frameworks is therefore essential to fully resolve this complexity. A key challenge has been that many studies inadvertently treat tDC^lo^, tDC^hi^, CD11b^−^ tDC2s, and tDC2s as separate entities rather than stages of a single differentiation trajectory, often assigning them distinct names across publications (Table 1). This issue largely stems from the widespread reliance on biaxial gating strategies with a limited set of surface markers, which can artificially fragment what is a continuous population. Given that tDCs are now recognized as forming a developmental continuum—distinct from canonical pre‐cDC2s—it becomes important to analyze them within a unified differentiation pathway.

### 9.1 Overview of cDC2 Subsets: Simplifying an Evolving Classification With Overlapping Nomenclature

**FIGURE 3:**
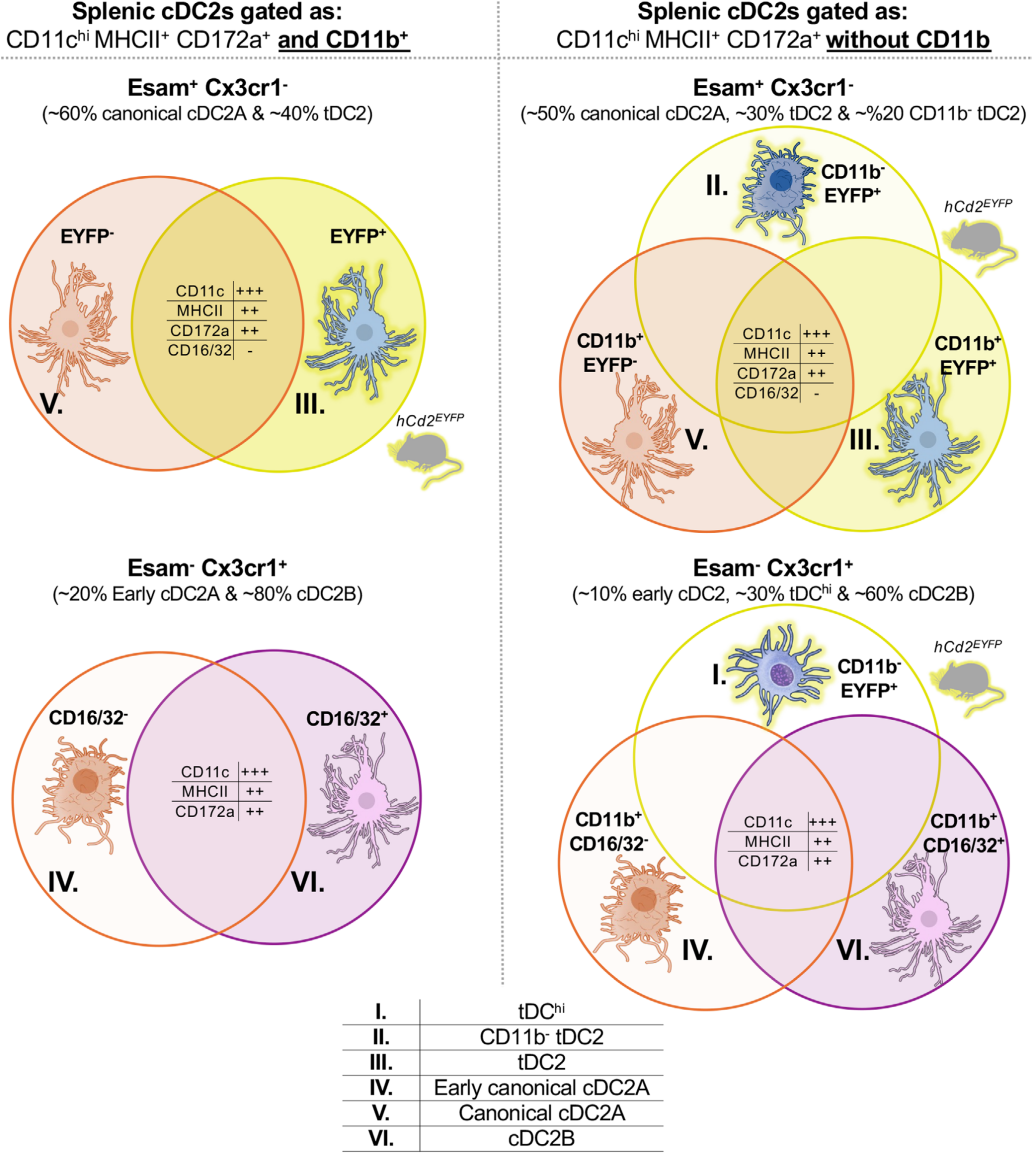
Mixture of cells in flow cytometry gating strategies with or without inclusion of CD11b staining. Two main gating strategies are commonly used in the literature for identifying mouse splenic cDC2s, which differ primarily in whether CD11b is included. When CD11b is included (left panels), tDCs and CD11b^−^ tDC2s are excluded, such that only tDC2s appear within the Esam^+^ Cx3cr1^−^ gate. In this case, only lineage‐tracing models can definitively distinguish tDC2s from canonical cDC2As. In contrast, when CD11b is omitted (right panels), tDC^hi^ cells fall within the Esam^−^ Cx3cr1^+^ gate and CD11b^‐^ tDC2s within the Esam^+^ Cx3cr1^‐^ gate. In both approaches, the Esam^−^ Cx3cr1^+^ gate contains early canonical cDC2As, which may be distinguished from cDC2Bs by their lack of CD16/32 expression. Tables summarize shared marker expression across populations. Cells labeled using the *hCD2*‐Cre x*Rosa* ^LSL‐EYFP^ (hCD2^EYFP^) lineage‐tracing model are underlined in yellow. Elements of this figure were created using resources from the NIH BioArt Source.

In mice, early work divided splenic cDC2s into two populations based on their dependency on Notch2 signaling and expression of Esam and Cx3cr1: Notch2‐dependent Esam^+^ Cx3cr1^−^ cDC2s, and Notch2‐independent Esam^−^ Cx3cr1^+^ cDC2s. The latter share some features with monocytes and are associated with pro‐inflammatory responses and Th17 polarization [33, 93, 94]. This dichotomy was later confirmed by Brown et al., who used T‐bet reporter mice to label Esam^+^ Cx3cr1^−^ cDC2s (termed cDC2A) and to distinguish them from unlabeled Esam^−^ Cx3cr1^+^ cDC2s (named cDC2B)—an observation reaffirmed by Minutti et al. [34, 36]. Notably, the cDC2B subset described by Brown included a minor contamination with RORγt‐expressing tolerogenic DCs [102], but overall, the definitions remain consistent with the original Notch2‐dependent and ‐independent cells. It is important to emphasize that Reizis', Murphy's and Brown's studies consistently included CD11b in their gating strategies, a practice critical for avoiding misclassification of tDC^hi^ cells (CD11b^−^ Cx3cr1^+^) as cDC2B (CD11b^hi^ Cx3cr1^+^) (Figure 3). This distinction is essential when integrating tDCs into this framework.

A similar dichotomy exists in humans. Initial scRNA‐seq studies by Villani et al. identified two major subsets within cDC2s: DC2s and DC3s [6], a division later confirmed by others [103]. DC3s express some monocyte‐associated markers such as CD14 and are pro‐inflammatory. Developmentally, DC3s appear to arise independently of common DC progenitors (CDPs), instead deriving via a GM‐CSF–dependent route from monocyte‐DC progenitors (MDPs) [103, 104, 105]. Parallel studies suggest that DC3s correspond to mouse Notch2‐independent Esam^−^ Cx3cr1^+^ cDC2B [32], although this alignment remains under discussion by some authors suggesting that cDC2B and DC3 are different subsets [36]. Nevertheless, whether referred to as cDC2B or DC3s, these cells share multiple features: high CD16/32 expression; partial (~10%–30%) labeling by monocyte lineage‐tracing models (e.g., *Lyz2*‐Cre and *Ms4a3*‐Cre); a transcriptomic profile including monocyte genes; dependence on Klf4 for development; and robust cytokine production with strong Th17‐polarizing potential, at least in vitro [30, 32, 33, 34, 36]. Altogether, these data support the existence of a population of proinflammatory DCs in both mouse and human, bearing traits of both cDC2s (CD11c^+^ MHCII^+^ CD11b^+^ CD172a^+^) and monocytes (Cx3cr1^+^ Ccr2^+^ CD14^+^ CD11b^+^). To minimize confusion, we refer to these cells in mouse spleen as cDC2B hereafter (Table 2).

Beyond this dichotomy, our recent work has uncovered additional developmental diversity within Notch2‐dependent cDC2As. As described earlier, two independent routes converge on the cDC2A pool expressing CD11b^+^ Esam^+^ Cx3cr1^−^. The first is the canonical pathway, proceeding through a CD115^+^ pro‐cDC → pre‐cDC2s → early cDC2A → canonical cDC2As in a Klf4‐dependent manner [30, 54, 55]. The second originates from tDCs, which develop from pro‐pDC in a Tcf4‐dependent manner and progress through tDC^lo^ → tDC^hi^ → CD11b^−^ tDC2 → tDC2 [17]. When considered alongside cDC2Bs, these findings support a model in which the murine spleen contains three developmentally distinct cDC2 subpopulations (Figure 2): tDC‐derived tDC2s (CD11b^+^ Esam^+^ Cx3cr1^−^); pre‐cDC2‐derived canonical cDC2As (CD11b^+^ Esam^+^ Cx3cr1^−^); and Notch2‐independent cDC2Bs (CD11b^+^ Esam^−^ Cx3cr1^+^).

These three developmental routes have been recently confirmed by Zhu et al. [11], though with some differences in nomenclature and interpretations. For example, their classification referred to tDC^lo^ as “pre‐DC2A” and tDC^hi^ as “DC2A,” while pre‐cDC2s and early cDC2A were termed “pre‐DC2B,” and “DC2B,” respectively (Table 1) [11]. This nomenclature compresses the continuum of tDCs into static categories and does not explicitly account for terminally differentiated tDC2s and canonical cDC2As (Table 1), which are far more abundant than their intermediates and represent the principal population analyzed in functional studies. Omitting the terminally differentiated Esam^+^ Cx3cr1^−^ cDC2A pool in the nomenclature risks misrepresenting functional data and introducing inconsistencies across studies, which may ultimately hinder translational applications.

Another key point of divergence concerns the developmental relationship between tDCs and pDCs. Zhu et al. argued against such a link based on the absence of *Klf4* ^EGFP^ expression. However, while TF expression can provide useful clues, it is the functional requirement—not mere expression—that most robustly defines lineage affiliation. Klf4 is dispensable for both tDC and pDC development [10, 11, 30], whereas both cell types require Tcf4 [8, 11, 17]. Moreover, Klf4 can be upregulated in pDCs during activation [42], supporting the view that this TF is dynamically regulated within the pro‐pDC/pDC/tDC lineage. The shared dependence on Tcf4, together with additional developmental and molecular features described in Section 5, provides compelling evidence for a common ontogeny between pDCs and tDCs.

### 9.2 Resolving the Nomenclature of the tDC Lineage and Its Relationship to cDC2s

As described earlier, tDCs follow a differentiation trajectory that advances from tDC^lo^ to tDC^hi^, to CD11b^−^ tDC2s, and culminates in fully differentiated tDC2s expressing CD11b^+^ Esam^+^ Cx3cr1^−^ (Figure 2). Each intermediate has been labeled differently across studies, usually as discrete populations rather than within a continuum (Table 1). Here, we align our classification with other nomenclatures to facilitate comparison and highlight the minimum set of markers required to reliably distinguish tDCs and their progeny from other DCs (Figure 3).

tDC^lo^ represents the earliest recognizable stage along the tDC‐to‐tDC2 differentiation axis. These cells are present in both circulation and spleen and phenotypically resemble pDCs, exhibiting higher expression of Siglec‐H and Ly6C but lower CD11c. Due to these traits, tDC^lo^ has also been referred to as “*Zbtb46* ^GFP+^ pDC‐like” cells [9, 10, 27], Siglec‐H^+^ Ly6C^+^ uncommitted progenitors [17, 55], and more recently Siglec‐H^+^ pre‐cDC2A [36] and Siglec‐H^+^ CD115^−^ pre‐DC2As [11]. Notably, all these definitions appear to encompass a fraction of tDC^lo^ as shown by pro‐pDC/pDC/tDC lineage‐tracing models, given the variable levels of *Zbtb46* ^GFP^, Siglec‐H and CD8 among these cells [8, 17].

tDC^hi^ cells represent the next step in this trajectory. These cells have lost expression of Siglec‐H and other pDC markers, together with reduced expression of Tcf4. However, they differ from cDC2s in key aspects: they express intermediate levels of MHC‐II and CD172a, and—critically—higher levels of Cx3cr1 while lacking CD11b and Esam. Because of this phenotype, tDC^hi^ can easily be mistaken for Esam^−^ Cx3cr1^+^ cDC2Bs and early canonical cDC2As if CD11b is not included in the gating strategy (Table 1 and Figure 3). Several studies illustrate this problem. Rodrigues et al. concluded that tDC^lo^ (gated as pDC‐like cells) differentiate into cDC2Bs and not cDC2A‐like cells, most likely due to the absence of CD11b in their gating strategy [10]. A subsequent study using a *Cd300* lineage‐tracing model reported that pro‐pDCs differentiate into both cDC2As and cDC2Bs, again reflecting the capture of two intermediate snapshots of the tDC‐to‐tDC2 trajectory—tDC^hi^ and tDC2s—without CD11b‐based discrimination [31]. Minutti et al. classified tDC^hi^ as CD8α^+^ cells to distinguish them from early canonical cDC2As and cDC2B [36]. However, we have shown that about one‐third of tDC^hi^ express CD8α [8, 17], indicating that CD8α^−^ tDC^hi^ cells were likely misclassified into other populations [36]. Zhu et al. attempted to separate tDC^hi^ from early cDC2As based on their lower expression of CD11b [11]. However, this strategy overlooked the fact that both populations are transient stages that ultimately differentiate into CD11b‐expressing tDC2s and canonical cDC2As. Moreover, it cannot conclusively separate tDC^hi^ from CD11b^−^ tDC2s, thereby collapsing two stages of differentiation with potentially different functions [11]. Together, these examples underscore the need to use a combination of markers for cDC2 classification—and, critically, to include CD11b as a defining marker (Figure 3).

Canonical pre‐cDC2s, in contrast, follow a parallel but independent trajectory (Figure 2): canonical pre‐cDC2s → early cDC2As (CD11b^+^ Cx3cr1^+^ Esam^−^ CD16/32^−^) → canonical cDC2As (CD11b^+^ Esam^+^ Cx3cr1^−^). Based on their phenotype, early cDC2As likely overlap with cDC2Bs. In the spleen, CD16/32 expression seems to help distinguish these two populations, as early cDC2As are CD16/32^−^ (Figure 3 and Table 1) [32, 36]. However, whether CD16/32 expression can reliably distinguish these cells outside the spleen remains unclear. Notably, early cDC2As were recently renamed DC2B by Zhu et al. [11], thereby recycling the “A” and “B” terminology in a different developmental context. As discussed above, early cDC2As represent a transient stage of pre‐cDC2 differentiation into Notch2‐dependent canonical cDC2As and therefore should not be treated as the final stage of this trajectory.

Altogether, the recycling of “A” and “B” designations to describe either intermediates or terminal populations has added considerable confusion. This ambiguity hinders clear comparisons of the tDC and canonical cDC2A lineage contributions across studies. Importantly, and as described in this manuscript, tDCs follow a continuum of differentiation. Collapsing this continuum into static categories obscures how functions and TFs are dynamically regulated along the trajectory—for example, Klf4 and Irf4 act at distinct steps—relationships that only become clear in the context of the full progression from pDC‐like to cDC2‐like states (Figure 2).

## 10 Conclusion and Remaining Questions

Over the last 8 years, tDCs have emerged as a distinct and functionally relevant subset within the DC compartment. Initially described as cells with atypical surface markers, tDCs have now been identified across species, traced developmentally to a progenitor shared with pDCs, and defined by a unique transcriptional and functional profile. Although they exhibit a capacity to differentiate into tDC2s, accumulating evidence demonstrates that tDCs are not merely developmental intermediates. Rather, they constitute a bona fide immune cell population, functionally and transcriptionally distinct from both pDCs and cDCs. While tDCs share pathogen‐sensing machinery with pDCs, their function diverges significantly: tDCs lack IFN‐I production but compensate with robust IL‐1β secretion following pathogen sensing, positioning them as early mediators of innate inflammation. At the same time, tDCs exhibit features of cDC2s: they process antigen, mature upon stimulation, and prime naïve CD4^+^ T cells. This functional duality is further refined across their developmental continuum—from tDC^lo^ to tDC^hi^ to CD11b^−^ tDC2s to tDC2s—highlighting their progression from inflammatory sensors to professional antigen‐presenting cells.

Despite these conceptual advances, several mechanistic questions remain. The transcriptional circuits governing tDC development, activation, and differentiation into tDC2s are only partially understood. Identifying the molecular cues that drive IL‐1β production, modulate antigen presentation, and regulate their differentiation trajectory will be essential for clarifying tDC function across tissues and disease contexts. Equally critical is defining the division of labor between tDC2s and canonical cDC2As—if such a division exists—particularly across tissues, during immune responses, and over the course of aging. The development of subset‐specific reporter models, lineage‐tracing systems, and refined depletion tools will be instrumental in addressing these questions in vivo.

As mentioned in the introduction, tDCs exemplify how a discrete immune subset can exist in multiple, temporally regulated cell states—each defined by specific transcriptional programs and functional capacities in homeostasis. Acknowledging this continuum is essential not only for accurately interpreting tDC biology but also for avoiding the fragmented nomenclature that has historically obscured their unified identity. tDCs therefore call for a more flexible conceptual framework for DC biology—one that integrates both developmental origin and context‐dependent functional programming.

Viewing DC subsets as dynamic, multi‐state programs shaped by both lineage and environment may apply beyond tDCs. Indeed, pDCs also appear capable of functionally reprogramming in response to environmental cues [27, 42, 67, 106]. This form of reprogramming—marked by the acquisition of TFs and effector functions characteristic of other DC subsets—goes beyond classical maturation and instead represents a deeper shift in cellular identity. In tDCs, such transitions likely occur as subtle, steady‐state changes influenced by tonic environmental inputs; in contrast, pDCs may undergo transition only in response to strong inflammatory stimuli such as high TNF levels in the absence of IFN‐I [42]. This flexibility in identity may be a unique feature of the pro‐pDC/pDC/tDC lineage—or may also apply to cDCs. For example, cDC1s have been shown to convert into cDC2‐like cells through manipulation of key TFs [107]; however, whether cDC1‐to‐cDC2 reprogramming occurs under physiological conditions remains unknown. Understanding the principles that govern these cell‐state transitions will not only refine our understanding of immune regulation but also open the door to new therapeutic avenues—including the reprogramming of DC identity in cancer, infection, and autoimmunity [108, 109].

## Conflicts of Interest

J.I. serves on the scientific advisory board of Immunitas Therapeutics; this affiliation is unrelated to the present work. The remaining authors declare no competing interests.

## Data Availability

No new data were generated or analyzed in support of this review.
